# Impact of Daily Thermocycles on Hatching Rhythms, Larval Performance and Sex Differentiation of Zebrafish

**DOI:** 10.1371/journal.pone.0052153

**Published:** 2012-12-20

**Authors:** Natalia Villamizar, Laia Ribas, Francesc Piferrer, Luisa M. Vera, Francisco Javier Sánchez-Vázquez

**Affiliations:** 1 Department of Physiology, Faculty of Biology, Regional Campus of International Excellence “Campus Mare Nostrum”, University of Murcia, Murcia, Spain; 2 Institut de Ciències del Mar, Consejo Superior de Investigaciones Científicas (CSIC), Barcelona, Spain; Vanderbilt University, United States of America

## Abstract

In the wild, water temperature cycles daily: it warms up after sunrise, and cools rapidly after sunset. Surprisingly, the impact of such daily thermocycles during the early development of fish remains neglected. We investigated the influence of constant *vs* daily thermocycles in zebrafish, from embryo development to sexual differentiation, by applying four temperature regimens: two constant (24°C and 28°C) and two daily thermocycles: 28:24°C, TC (thermophase coinciding with daytime, and cryophase coinciding with night-time) and 24:28°C, CT (opposite to TC) in a 12:12 h light:dark cycle (LD). Embryo development was temperature-dependent but enhanced at 28°C and TC. Hatching rhythms were diurnal (around 4 h after lights on), but temperature- and cycle-sensitive, since hatching occurred sooner at 28°C (48 hours post fertilization; hpf) while it was delayed at 24°C (96 hpf). Under TC, hatching occurred at 72 hpf, while under CT hatching displayed two peaks (at 70 hpf and 94 hpf). In constant light (LL) or darkness (DD), hatching rhythms persisted with tau close to 24 h, suggesting a clock-controlled “gating” mechanism. Under 28°C or TC, larvae showed the best performance (high growth and survival, and low malformations). The sex ratio was strongly influenced by temperature, as the proportion of females was higher in CT and TC (79 and 83% respectively), contrasting with 28°C and 24°C, which led to more males (83 and 76%). Ovarian aromatase (*cyp19a*) expression in females was highest in TC and CT (6.5 and 4.6 fold higher than at 28°C, respectively); while anti-müllerian hormone (*amh*) expression in males increased in testis at 24°C (3.6 fold higher compared to TC) and particularly at 28°C (14.3 fold increase). Taken together, these findings highlight the key role of environmental cycles during early development, which shaped the daily rhythms in fish embryo and larvae, and ultimately influenced sex differentiation.

## Introduction

Seasonal and daily environmental changes caused by geophysical cycles (i.e., revolution and rotation of the Earth) have shaped the evolution of rhythmic physiological and behavioural processes in animals, which have developed biological clocks to anticipate cyclic events, such as alternations between night and day, and the subsequent changes in temperature [1]. In aquatic ectotherms, temperature entrainment has a crucial adaptive significance as the physical dynamic of the water creates a challenging ecosystem to which the organism must adapt in order to avoid thermal stress [2]. In the wild, water warms up after sunrise (thermophase) and cools down after sunset (cryophase). However, under artificial rearing conditions (e.g., fish farms), the natural environmental fluctuations are rarely considered. Currently, constant thermal environments are thought to maximize growth, but at the cost of reducing thermal tolerance and phenotypic diversity in a population (animals reared under cyclic conditions are more likely to survive environmental changes) [2].

In fish, temperature affects virtually all aspects of their behaviour and physiology, including (but not limited to) locomotion [3], foraging ability [4], growth [5] and sex differentiation [6]. There are three possible mechanisms of action of variations in ambient temperature on animal physiology: circadian entrainment, masking, and non-temporal effects. Thus, temperature cycles may act as the main entrainment factor even when light oscillations are also applied, as reported for the circadian rhythms of hatching and clock gene expression in *Drosophila* [7]. However, the effect of daily thermocycles on fish embryo and larvae development has been neglected, although there is evidence indicating that temperature cycles play an important role in the onset of the fish molecular clock. In fact, in zebrafish (*Danio rerio*) embryos reared in the absence of light, the temperature cycles entrained their circadian rhythms of clock gene expression [8].

Fish account for more than half of all known vertebrate species and zebrafish is a well known model system for the study of the circadian clock [9]. Zebrafish has small, transparent embryos with a fast development process (24 h from one fertilized cell to a recognizable vertebrae embryo) that can be observed non-invasively under a microscope [10]. As in most ectotherms, temperature is such a strong synchronizer that even daily thermocycles of narrow range (1–2°C) are capable of entraining their circadian rhythms of locomotor activity [11]. Adult zebrafish have a marked activity rhythm, which starts to oscillate robustly early in larvae [1]. Although zebrafish has been described as a diurnal species [12], recent studies have found that it is capable of displaying either diurnal or nocturnal behavioural rhythms depending on the rearing conditions (i.e., feeding and temperature cycles) [13], [14].

Water temperature may have irreversible effects during sensitive periods of early development in fish. For instance, sex determination can be temperature-dependent, so that temperature determines whether an individual will develop as a male or as a female [15]. Recently, an epigenetic mechanism linking high temperature and masculinization through hypermethylation of the promoter of the gonadal aromatase gene (*cyp19a*) has been proposed in the European sea bass, a species with polygenic sex determination with environmental influences [16]. The zebrafish is a gonochoristic species following what is known as the undifferentiated type, whereby all individuals start ovarian differentiation around 10 dpf. Sometime near 20 dpf, about half of them stop ovarian differentiation and engage in male differentiation while the other half continue with ovarian differentiation [17]. In addition, thermal effects on sex differentiation are superimposed on an otherwise essential genetic sex determination system [6]. However, although it is agreed that zebrafish has no heterochromosomes, their sex determining mechanism is far from clear, except that the genetic component (as opposed to the environmental one) is of some relevance. Sex in zebrafish has been proposed to be determined by female-dominant genetic factors compatible with a ZZ/ZW chromosomal system [18]. However, a different study using three different zebrafish strains and a single nucleotide polymorphism (SNP) -based linkage map, found two chromosomes governing sex as a complex trait rather than a XX/XY or a ZZ/ZW system [19]. Later, a study based on high throughput sequencing of more than 30.000 SNPs revealed that sex-associated locus in zebrafish was found in two other different chromosomes [20]. Furthermore, a recent research using classical breeding experiments and selection demonstrated that zebrafish sex has a genetic basis and proposed a polygenic sex determining mechanism [21]. Likewise, the effects of temperature on zebrafish sex differentiation remain unclear as just few studies have been carried out [22], [23], although a masculinizing effect of high temperature is suspected [6]. As in other teleosts, gonadal aromatase (*cyp19a*) and the anti-müllerian hormone (*amh*) have been found to play a key role during sex differentiation. On the one hand, *cyp19a* controls sex differentiation in teleosts by regulating estrogen synthesis and so ovarian development [24], [25]. On the other hand, zebrafish *amh* has a sexually dimorphic expression pattern as it has been detected in testis but not in ovaries, being antagonistic of *cyp19a* [26]. Thus, the effects of temperature on zebrafish sex ratios are far from clear. Furthermore, the effects of daily thermocycles on sex differentiation remain completely unexplored.

Therefore, since environmental cycles have strong effects during sensitive periods of early development in fish, the aim of this study was to investigate the effect of water temperature (constant *vs* daily cycling) on embryo somitogenesis, hatching rhythms, larval growth, behaviour and sex differentiation of zebrafish.

## Materials and Methods

### Animal Rearing Conditions

The present research was conducted at the Chronobiology laboratory located at the Faculty of Biology, University of Murcia (Spain). Adult zebrafish (*Danio rerio*) of heterogeneous wild-type stock (standard short-fin phenotype) were obtained from a local commercial distributor and housed for 6 months at the Chronobiology laboratory according to standard methods [27]. Six groups of sexually mature zebrafish (2 females: 4 males per group) were used as broodstock. Fertilized eggs from spontaneous spawnings were collected and pooled together in the morning, within 2 h of laying and were distributed into 85×10 mm sterile Petri-dishes filled with embryo medium for 5 days (35 embryos per Petri dish). After this period, larvae were transferred into 2.5 L nursery net cages (3 Petri dishes per cage, N = 105 larvae) (SERA GmbH, Germany). Petri-dishes and cages were both kept in 9 L thermostat-controlled glass aquaria. For each experimental group two aquaria were used (N = 210). The photoperiod was 12 h of light:12 h of darkness (LD) of neutral white light (1.70±0.03 µmoles m^−2^. s^−1^, which corresponds to 91.8±3.6 lux; Flexible LED stripes, Superlight Technology Co., Ltd, China). From 7 days post fertilization (dpf) onwards, larvae were fed to satiation twice a day with powdered food (JBL Novo Tom, JBL GmbH & Co. KG, Germany). From 15 dpf onwards, artemia nauplii (JBL GmbH & Co. KG, Germany) were also supplied (once a day), and from 20 to 90 dpf powdered food was replaced by granulated food (also twice a day) (Biogran Small, PRODAC, Italy).

The aforementioned procedure was repeated in order to obtain two independent biological replicates and to confirm the sex ratio results. Therefore, two different batches of eggs from the same broodstock groups were used.

All zebrafish husbandry and experimental procedures were approved by the European Convention for the Protection of Animals used for Experimental and Scientific Purposes (ETS N° 123, 01/01/91). The experimental protocol was previously authorized by the Spanish National Committee on Animal Welfare (Law 32/2007) and the Bioethical Committee of the University of Murcia.

### Experimental Procedure

**Figure 1:**
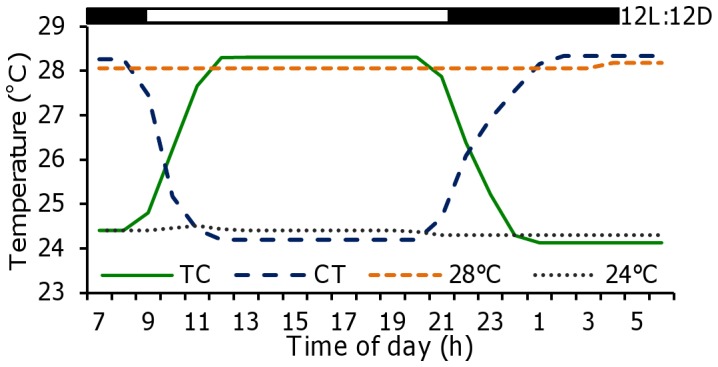
Experimental thermocycles and constant temperatures. 24:28°C thermophase:chryophase (TC), 28:24°C (CT) and constant treatments of 24°C and 28°C. The bar at the top represents the photocycle (12:12 LD).

From 0 to 42 dpf, embryos and larvae were reared in one of the following four temperature regimes (Figure 1): low constant temperature of 24.3±0.1°C, high constant temperature of 28.1±0.1°C, a thermocycle of 28.3:24.1±0.1°C (thermophase:cryophase, TC), where the thermophase coincided with the light period (lights on: 09:00 h, lights off: 21:00 h) and the cryophase coincided with the dark period and an inverted thermocycle of 24.2:28.3±0.1°C (CT), where the thermophase coincided with the dark period and the cryophase coincided with the previous light period. To test the endogenous control of hatching rhythms, three temperature regimes were used with cycling temperature (TC), constant 24°C or constant 28°C, under constant light (LL) or constant darkness conditions (DD). Water temperature was modified by means of water heaters (100 W, Askoll, Italy) or chillers (Aqua Medic Titan 500 GmbH, Germany), controlled by an electronic timer (Bachmann GmbH & Co, Germany). Temperature was recorded every 10 minutes by means of an underwater data logger (HOBO PENDANT® Onset Computer Corporation, Massachusetts, USA) placed in each aquaria. From 43 to 150 dpf, all fish were separately kept under 28.0±0.5°C.

### Data Collection

All observations were made in live embryos and larvae and no mortalities were registered during the sampling procedures. Embryo development was assessed every two hours by counting the number of somites from 10 to 30 h post-fertilization (hpf, 60 embryos per temperature group) [28]. Hatching was determined by counting the number of newly hatched larvae from 48 to 100 hpf, also every two hours. Growth was recorded by measuring the total length of 60 larvae per temperature group from 2 dpf and every fifth day until 42 dpf. The number of larvae with physical malformations was recorded throughout the experiment. Embryos and larvae were observed and measured using a digital camera mounted on a dissecting microscope. To ensure the survival of the embryos and larvae, the samples were collected (30 per aquarium) and placed into a new Petri dish filled with the same water of their corresponding temperature group. Samples were quickly photographed and placed back into each aquarium. The digital images were analysed to obtain standard measurements using image processing and analysis software (Leica Microsystems Imaging Solutions Ltd, Cambridge, UK). Survival was determined at 42 dpf, by counting the number of remaining larvae with respect to the initial number of hatched larvae of each temperature group.

For behavioural recording, at 3 and 10 dpf, 60 larvae from each temperature group were video recorded for 1 h (Sony Handicam, DCR-SR55E) during the middle of the dark phase (MD, 03:00 h) and the middle of the light phase (ML, 15:00 h). For all video recordings, infrared lights (λ = 880 nm) were used as the principal illumination source. Videos were analysed to count the number of active/inactive larvae (swimming/motionless) and to calculate the swimming activity of each temperature group during the MD and ML phases. Locomotor activity analysis was performed by means of specialised “Fishtracker” software designed and validated by the Computer Vision Research Group of the University of Murcia [29]. The software tracked and updated the position (X, Y) of each fish every second, creating a data worksheet from which mean activities were calculated and compared (ML vs MD activity within each group and among temperatures groups).

To assess sex differentiation at 42 dpf, 70 post-larvae per temperature group were killed in iced water and fixed in Bouin’s fixative for 48 h. Specimens were dehydrated in alcohol, cleared in xylol, and infiltrated and embedded in paraffin. Serial sections (6–7 µm) were stained with haematoxylin-eosin (H&E) and examined by light microscope. Gonadal cell stages were classified according to previous morphological studies on gametogenesis in female and male zebrafish [30], [31].

Gene expression analysis were performed at 90 dpf, using 30 fish randomly collected from each temperature group, anesthetized and euthanized on ice. Gonads were removed for sex ratio assessment. Then gonads of 10 males and 10 females per experimental group were individually homogenized in Trizol reagent (Invitrogen, Madrid, Spain) using a tissue homogenizer (POLYTRON^®^, PT1200, Kinematica, Switzerland). Total RNA concentration was determined by spectrometry (Nanodrop^®^ ND-1000, Thermo Fisher Scientific Inc., Wilmington, DE, USA), and 1 µg was treated with DNA Wipeout (Qiagen, USA) to prevent genomic DNA contamination. cDNA synthesis was carried out with Quantiscript Reverse Transcriptase and Primer Mix (Qiagen, USA) in a 20 µl reaction volume. Quantitative PCR was performed using the StepOnePlus Real-Time PCR System (Applied Biosystems, Foster City, CA, USA) with SYBR-green-primer-master mix according to the manufacturer’s recommendations. Amplification was carried out with the following cycle conditions: 15 minutes at 95°C, then 40 cycles of 15 s at 95°C and 30 s at 60°C. The final volume of the PCR reaction was 20 µl: 5 µl of cDNA, 10 µl of the qPCR Master Mix and 5 µl of forward and reverse primers.

The primers for *cyp19a* were designed with Primer Express Software (Applied Biosystems, Foster City, CA, USA), (F) 5-TGCTGGCCATCAGACACCAT-3 and (R) 5-CAGATGAACCGACAGTAGGAGACAA-3. The primer sequences for *amh* were retrieved from the literature, (F) or 5- GGGTGTGCATGCTACAGAAGAT- 3 and (R) 5-CTCAGAAATGCAAACAGTCTGTGT-3 [32]. The amplification efficiency, specificity of primers and amount of cDNA/sample were tested by the standard-curve method. The relative expression of all genes was calculated by the 2^−ΔΔCT^ method [33], using *D. rerio ef1α* (F): 5-CTGGAGGCCAGCTCAAACAT-3 and (R): 5-ATCAAGAAGAGTAGTACCGCTAGCATTAC-3(ENSDART00000023156) as the endogenous reference [34].

By 150 dpf, the remaining fish (N for 24°C = 60, for 28°C = 78, for TC = 69 and for CT = 36 fish) were externally identified as males or females following the dimorphic features described for this species [35]. This sampling was used to confirm the final sex ratio among treatment groups.

### Statistcial Analysis

To test for statistical differences among treatments, all data were first tested for normality with the Kolmogorov–Smirnov’s test and all were found to be normal. Then, data were analysed by one-way analysis of variance (ANOVA) followed by Tukey’s multiple range test to determine significant differences. The Student’s *t*-test was performed on data of larval activity to compare the number of active larvae and swimming activity of each group in ML and MD. To find statistical differences among treatments in the proportion of malformations and in sex ratios, data were arcsine transformed before being analysed using one-way ANOVA. Statistical analyses were carried out using the software SPSS 15.0 (SPSS Inc.). *P* values <0.05 were considered statistically significant. All data are expressed as mean±S.E.M.

## Results

### Embryo Development and Hatching Rhythms

**Figure 2:**
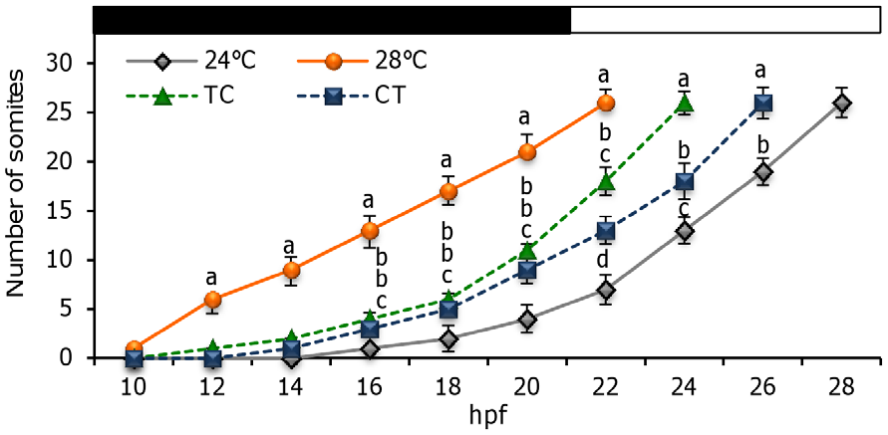
Zebrafish somitogenesis as a function of constant and cycling rearing temperature regimens. Black and white bars represent the night and day periods of the 12:12LD cycle, respectively. hpf: hours post fertilization. Values are expressed as mean±SEM. Different letters indicate statistical differences among groups (ANOVA, *P*< 0.05, N = 30 per temperature group). Abbreviations as in Figure 1.

The effect of temperature was clearly observed during zebrafish embryogenesis as somitogenesis occurred first in larvae under 28°C, with a regular/constant formation of somites during the dark phase, ending at 22 hpf. In contrast, in the CT and 24°C groups, somitogenesis took 4 and 6 hours longer to be completed (until 26 and 28 hpf, respectively) while in the TC group it took place at 24 hpf. Somite formation was not regular in the TC, CT or 24°C larvae since it was slower during the dark phase (with a mean of 2.1±0.3 somites being formed every two hours) than during the light phase (6.2±0.5 somites 2 h^−1^, *P*<0.05) (Figure 2).

**Figure 3:**
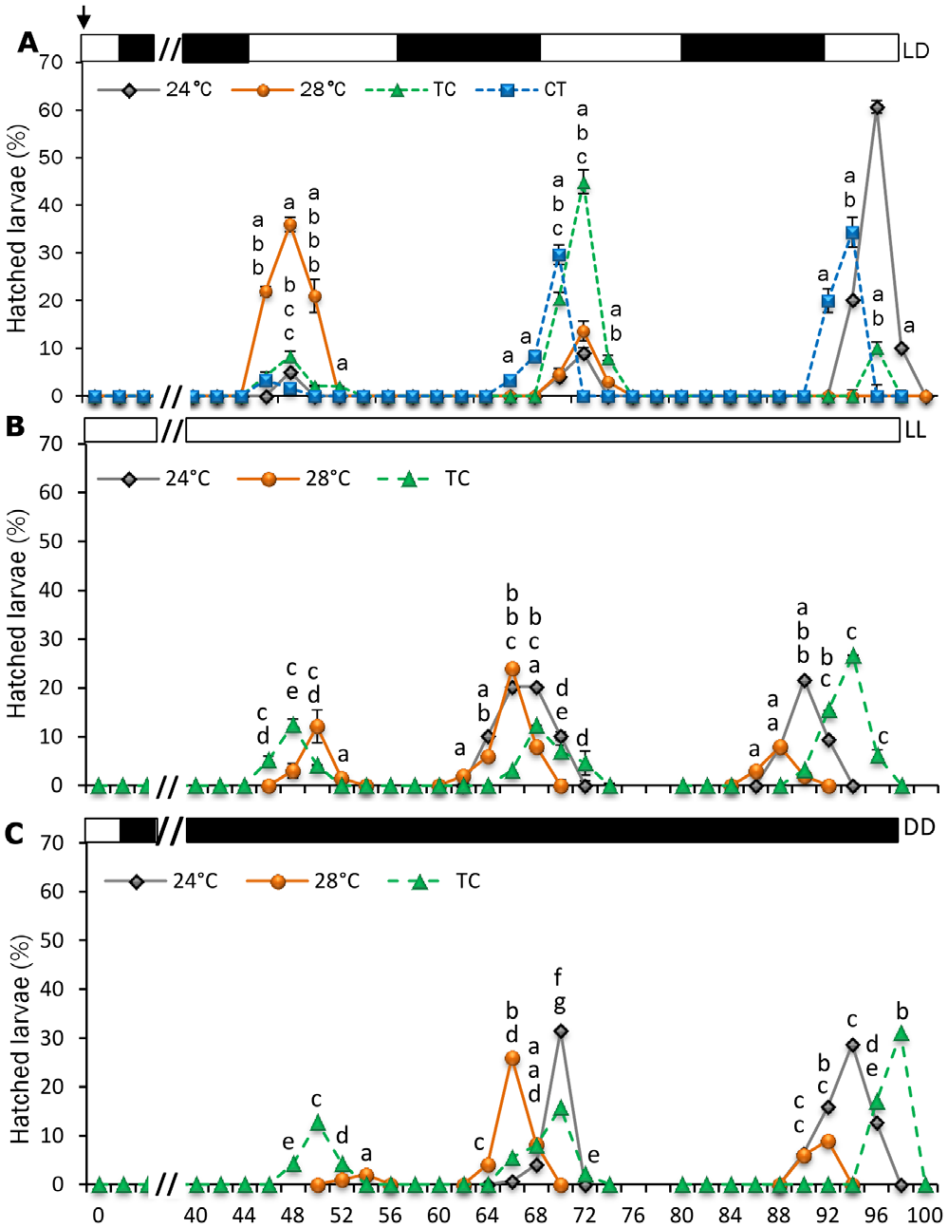
Effect of temperature on zebrafish hatching rhythms. Fertilised eggs were incubated under LD (A), LL (B) and DD (C). Values are expressed as the percentage of newly hatched embryos observed every two hours, during three consecutive days (44 – 100 hpf). White and black bars represent the day and night phases, respectively. The arrow represents the time of fertilization. Data are expressed as mean±SEM. Different letters indicate statistical differences among groups at the specific sampling time (ANOVA, *P*<0.05, N = 30 per temperature group). Abbreviations as in Figure 1.

A rhythmic pattern of hatching was apparently synchronized to the LD cycle, with a diurnal acrophase in all treatments regardless of temperature conditioning. Embryos that did not hatch at the beginning of the light phase at 2 dpf hatched 24 hours later, coinciding with the start of the next light cycle. Temperature marked the start of hatching since by 50 hpf 79.2±4.4% of larvae reared under constant 28°C were swimming free out of their chorion. At this time, 12.5±4.3% of larvae reared under TC had hatched, in contrast with only 5.6±3.6% and 5.3±0.4% of the CT and 24°C groups, respectively. By 3 dpf (76 hpf), all larvae from the 28°C group hatched as well as most of the TC larvae. In contrast, larvae under 24°C showed the lowest hatching rate (45.3±2.3%, *P*<0.05). The time of the first hatching peak was the same for all treatments by 48 hpf. However, by 3 and 4 dpf, the hatching peak of larvae under CT occurred 2 h before the rest of the groups (70 and 94 hpf) (Figure 3A). Under constant lighting conditions (LL and DD), hatching rhythms persisted with tau close to 24 h in all groups investigated. Under TC and 28°C, the number of hatchlings was distributed across three circadian cycles in constant conditions (LL and DD), whereas most embryos hatched within the first two cycles in LD.

### Larval Performance

**Figure 4:**
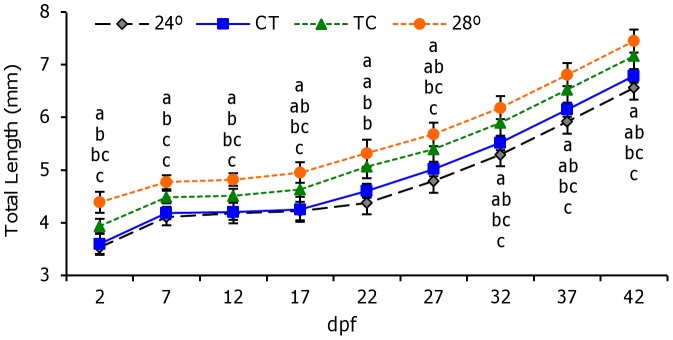
Influence of temperature on zebrafish larval development. Data are expressed as total length (mean±SEM) from 2 to 42 days post fertilization (dpf). Different letters indicate statistical differences among groups (ANOVA, *P*<0.05, N = 30 per temperature group). Abbreviations as in Figure 1.

Significant differences in growth were found among treatments from the beginning of the experiment. The greatest larval length was found in the 28°C group, followed by TC, while the lowest size was observed in larvae from the 24°C regimen (*P*<0.05). These differences were present throughout the experiment until 42 dpf (Figure 4).

**Figure 5:**
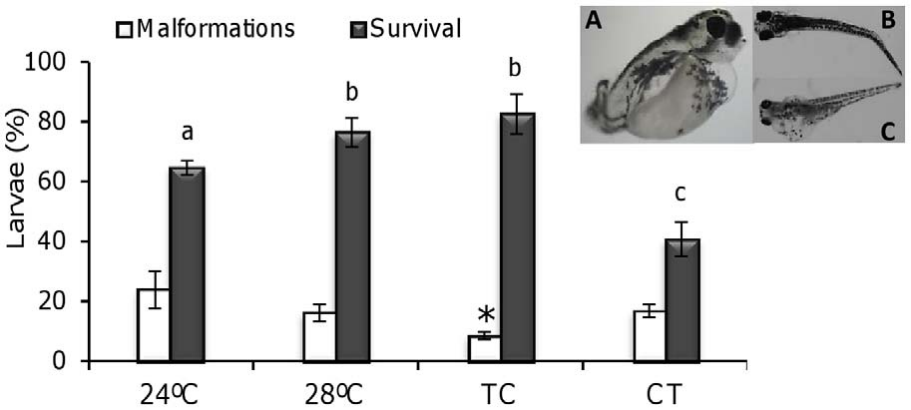
Malformations and survival of zebrafish larvae reared under constant and cycling temperatures. Observed malformations (0 to 42 dpf, N = 30 per temperature group) included delayed yolk sac resorption (**A**), spinal column curving (**B**) and pericardial edema (**C**). Asterisk (malformations) or different letters (survival by 42 dpf) indicate statistical differences among groups (ANOVA, *P*<0.05). Abbreviations as in Figure 1.

The main physical alterations observed in this study were bent and hook-like tails, spinal column curving, pericardial edema, retarded yolk sac resorption, and shorter body length. Malformations were found in all groups in 16.4±3.1% of the individuals (mean value of the four temperature groups) but they were significantly lower in the TC group, where malformations were only present in 8.5±1.3% of the larvae (*P*<0.05; Figure 5). By 42 dpf, the highest survival was also found in the TC group, with no statistical differences compared with the 28°C group (82.8±6.7% and 76.5±6.8%, respectively; *P* = 0.18). The lowest survival was found in larvae reared under the CT regimen (40.9±5.8%) (Figure 5).

### Activity

**Figure 6:**
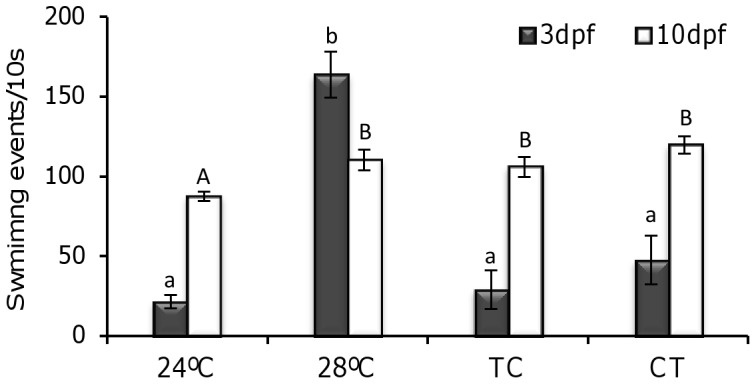
Locomotor behaviour of zebrafish larvae reared under different temperature groups. Swimming activity (number of events recorded every 10 seconds) was observed in 3 and 10 days post fertilization (dpf) larvae. Data is expressed as mean±SEM. Different lowercase (3 dpf) and uppercase (10 dpf) letters indicate statistical differences among temperature groups (Student’s t-test and ANOVA, *P*<0.05, N = 30 per temperature group). Abbreviations as in Figure 1.

Swimming activity was highest in the 28°C group by 3 dpf (Figure 6), with all larvae being active (swimming) during ML, in contrast with the low number of active larvae found in the 24°C group (1.3±0.3%, *P*<0.05) (Table 1). By 10 dpf, the number of active larvae was highest during ML in all groups with no significant differences being observed (P = 0.23).

**Table 1:** Influence of water temperature (constant vs daily thermocycles) on locomotor activity of zebrafish larvae.

	3 dpf				10 dpf			
	24°C	28°C	TC	CT	24°C	28°C	TC	CT
ML	1.3±0.3^*a^	100^*b^	61.3±1.4^*c^	30.6±4.0^*d^	100^*^	100^*^	100^*^	100
MD	32.3±3.1^a^	85.1±3.2^b^	2.5±0.1^c^	75.6±6.2^b^	63.2±4.1^a^	36.5±2.0^b^	22.1±2.1^c^	94.3±6.1^d^

Active larvae: percentage of larvae in motion (swimming) during ML and MD (mean±SEM, n = 60) in each group.

Asterisks represent significant differences found between ML and MD within each experimental group while lower case letters represent statistical differences among treatments (Student’s t-test and ANOVA, p<0.05).

### Sex Ratio and Sex Differentiation

**Figure 7:**
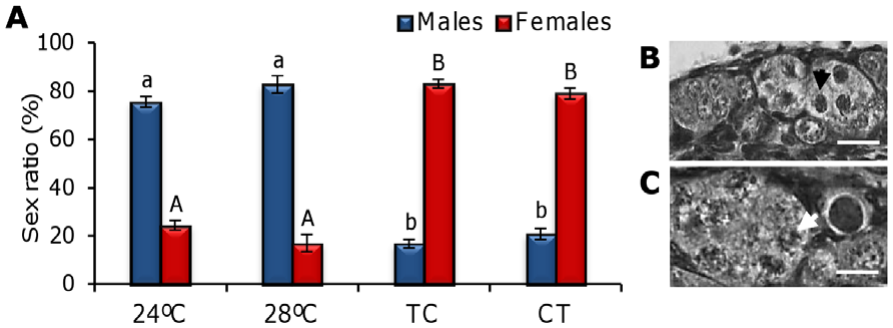
Sex ratio of zebrafish exposed to constant and cycling temperatures for 42 dpf. Values are expressed as mean percentage of males or females obtained at 42, 90 and 150 dpf (**A**) (mean±SEM). Different letters indicate statistical differences in the percentage of males (lowercase) and females (uppercase) found among groups (*P*<0.05). Photos: developing male gonad (**B**) with spermatogenic cells (black arrowhead) and female gonad (**C**) with clusters of oogonia (white arrowhead). Scale bar: 20 µm. (N of each group: 24°C = 160, 28°C = 178, TC = 169 and CT = 136). Abbreviations as in Figure 1.

The sex ratios were determined from two biological replicates at 42 dpf by histological analysis, by observation of the gonads upon dissection at 90 dpf, or by examination of the external dimorphic sex phenotype at 150 dpf. The total number of fish used for each group was: 24°C = 160, 28°C = 178, TC = 169 and CT = 136. In zebrafish larvae reared under 28°C and TC gonadal development by 42 dpf occurred earlier than in the other groups, as sex could be determined in most of the sampled fish (92%), in contrast with the CT and 24°C groups, where only 80% and 74% of the fish, respectively, could be histologically sexed. Sex ratio results revealed that fish under the thermocycles showed a significantly higher proportion of females (79.0±2.3% in CT and 83.0±1.8% in TC), whereas females accounted for just 24.4±2.1% and 17.0±3.5% of larvae exposed to the constant temperatures of 24°C and 28°C respectively (*P*<0.05) (Figure 7).

**Figure 8:**
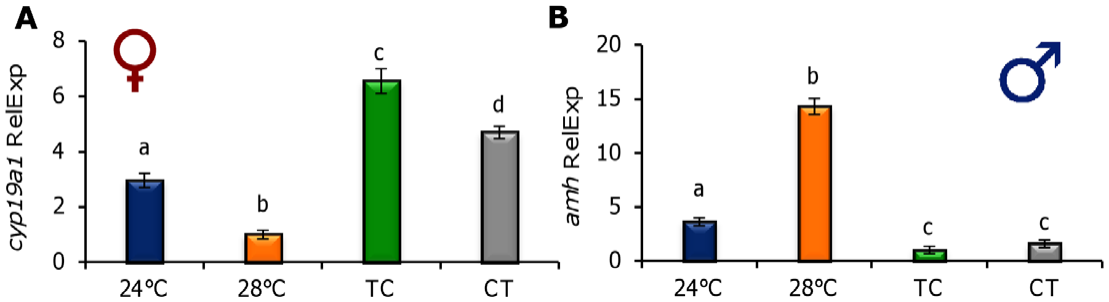
Effect of constant and cycling temperatures on gonadal sex determination genes. Relative expression of *cyp19a* (**A**) measured in the gonads of females, and *amh* expression (**B**) in males of 90 days post fertilization zebrafish. Different letters indicate statistical differences among groups (*P*<0.05). Data are expressed as mean±S.E.M. (n = 10 each sex). Abbreviations as in Figure 1.

Gene expression of *cyp19a* and *amh* was also affected by the temperature regimens, as in females *cyp19a* was overexpressed under the thermocycles (TC and CT), in contrast with the low levels found in the constant temperature groups (24°C and 28°C). In addition, *cyp19a* expression was significantly higher in the TC than in the CT groups (6.5 and 4.6 fold higher than in the 28°C group, respectively, *P*<0.05) (Figure 8A). In contrast, in males *amh* was overexpressed in the 28°C group followed by males kept under 24°C (14.3 fold increase and 3.6 fold higher compared to TC, respectively) (Figure 8B) but lower expression was found in the CT and TC groups (*P*<0.05).

## Discussion

Temperature has long been reported as a key factor affecting the behaviour and physiology of ectotherms in which body temperature depends on the thermal environment [36]. Our findings provide evidence for such exogenous control in accordance with a previous study, where 28.5°C was established as the optimal temperature for zebrafish embryo development and hatching rate [28]. On the other hand, endogenous mechanisms also play a role, as the fish circadian system comprises a multioscillatory system entrained by light (LEO), food (FEO) and temperature (TEO) cycles, although the coupling between them remains unknown [11]. While the period of circadian oscillators is temperature compensated, such that the period remains relatively constant over a physiological range of temperatures, circadian clocks can still be entrained by temperature cycles [8]. Somitogenesis is a rhythmically repeated process determined by a genetic oscillator known as the segmentation clock [37]. This sequential pattern of embryo development has been studied by many authors and its periodicity calculated in several species under a wide range of temperatures, and it has been found that somitogenesis frequency depends linearly on constant temperature [38], [39], [40]. Although the delay in larvae segmentation due to low temperatures is well known in zebrafish, the effect of daily thermocycles and their interaction with the photocycle had never being investigated. Our data revealed that the rate of segmentation is slower in CT than in TC, even though the mean temperature in both thermocycles was similar (26°C). Thus, the ultimate effect on somitogenesis is not only the temperature itself, but also the relationship between the daily rhythm of cell division and the thermo-/light-phase. Since light regulates the zebrafish cell cycle, so that cells enter the S phase during daytime [41], we hypothesise that the combined effect of high temperatures during the day (TC) enhances cell proliferation, while the reverse condition (lower temperature during daytime, CT) delays normal development.

Daily reproduction rhythms have recently been reported in diurnal and nocturnal teleost fish. The time of spawning depends on the daily pattern of behavior of the species (i.e., diurnal or nocturnal), such that spawning takes place during the light phase in diurnal zebrafish [42], during the dark phase in nocturnal species (e.g., Senegalese sole, *Solea senegalensis* [43] and European sea bass, *Dicentrarchus labrax* [44]), or during the day-night transition (e.g., gilthead seabream, *Sparus aurata* [45]). Our study demonstrates that both embryogenesis and hatching are also coincident with the active phase of zebrafish. Nevertheless, although hatching rhythms were strongly light-entrained, they were also temperature-sensitive, as hatching was advanced to 48 hpf in the 28°C group and delayed to 96 hpf in the 24°C group, while hatching occurred at 72 hpf in TC and was split into two peaks taking place at 70 hpf and 94 hpf in the CT group (Figure 3A). Interestingly, under LD hatching at night did not occur, which suggests that zebrafish embryos are light-sensitive and hatching is “gated”, so that it occurs during a specific time of day (window). Indeed, light detection has been observed in zebrafish embryonic cells as early as 5 hpf (gastrula stage) and this early ability to detect light has been linked with the rise of clock genes and transcripts implicated in DNA repair [46], [47]. A similar “gating” phenomenon of eclosion has been reported in insects [48], [49]. In *Drosophila*, hatching time is determined by the interplay between the embryo developmental state and the circadian clock [50]. The notion of gating implies there is one process that has a relatively constant rate and another process that gates the first one. Thus, presumably, embryos develop at a relatively constant rate (“developmental clock”) but hatching is limited to brief windows at 24-hour intervals. As reported in insects, it is likely that in zebrafish a clock-controlled mechanism measures development, so that those embryos that reach a certain developmental stage by a certain circadian phase hatch during the first available window or “gate”, while those that have not must wait until the next cycle. This hypothesis is supported by our data as hatching rhythms persisted in DD and LL (Figure 3B-C), indicating that gating is controlled by a circadian clock rather than by the LD cycle. It should be also noted that the circadian gating component can be entrained by the LD cycle (and apparently also by the thermocycles), but entrainment is not necessary for gating.

Studies on growth and development under cyclic temperatures are scarce in fish. There are a few reports on the acclimation of metabolism to cycling temperature conditions [51], [52]. So current knowledge is mostly based on results obtained under constant temperature regimens, where sub-optimal temperature conditions were found to increase the occurrence of fatal deformities and mortalities due to the stress caused by altered developmental timing [53]. In the present study, the total length of larvae in the 28°C group was always significantly higher than in the rest of the groups, while larvae kept at 24°C showed the slowest growth (Figure 4). Under TC/CT larvae performance was in-between, as did the mean temperature of these daily thermocycles (26°C). It is important, however, to distinguish between chronic and cyclic effects of temperature. It is interesting to note that the growth of larvae under TC and CT differed during the course of the study despite being exposed to the same mean temperature. As hypothesised for embryos, such differences in larvae development under TC and CT may be linked to the existence of daily rhythms in the endocrine somatotropic axis and the relationship between daily cycles of light and temperature. The expression of growth factors (GH, IGF-I, IGF-II) in fish has a daily rhythm synchronised to the LD cycle [54]. Furthermore, an early study with the largemouth bass (*Micropterus salmoides*), found that fish grew faster when maintained at high-low cycles of temperature and were fed at the onset of high temperature [55]. These results point to “optimal temperature cycles”, rather than optimal constant temperatures, to maximise growth performance and minimise malformations. In the present study, malformations were lowest while survival was highest under the TC regime (although there was no significant difference in survival compared to 28°C), in accordance with a previous study in which zebrafish maintained under a variable temperature displayed a higher temperature tolerance than fish maintained under a constant temperature [2].

Light environmental conditions during early development have drastic effects on fish larvae performance and behaviour [56]. Recently, a light-dependent switch from diurnal to nocturnal behaviour has been reported in flatfish larvae undergoing metamorphosis [57]. In zebrafish we observed many active larvae at ML by 3 dpf in the 28°C and TC groups, while in CT larvae were mostly active during MD. It has been suggested that LD cycles set the phase of the clock that controls zebrafish larval behaviour, with rhythmic activity synchronized to the LD cycle, with a diurnal acrophase as early as 2 dpf [58]. These results suggest that at this stage the daily thermocycle could be the main synchroniser. However, since in this research the activity of larvae was not recorded under constant light conditions, a masking effect of light can not be ruled out. In a previous study most adult zebrafish under constant light synchronised their activity rhythms to daily thermocycles (26:20°C) [59]. Moreover, when light and temperature cycles with different periods (25 h and 23 h period for the light and temperature cycles, respectively, i.e., conflicting zeitgebers) were applied, relative coordination between light and temperature could be observed. It appears that when light/dark and high/low temperature cycles are combined (TC), a stable phase and maximum amplitude can be observed in the rhythms, while when the two synchronizers are phase-shifted (CT), the phase of the rhythm is determined by either zeitgeber or by both, depending on the relative strength of each and the sensitivity of the species [60].

In fish, temperature-dependent sex determination (TSD) has been reported many times [61], [62], although it is not as common as was previously thought. Thus, many instances of sex ratio shifts observed at high temperatures are, most likely, the consequence of thermal effects on an otherwise predominantly system of genotypic sex determination (GSD) rather than proof of the existence of TSD [6]. In some species, the shift in sex ratios under laboratory conditions may occur at extreme temperatures, which are not normally found in the wild [15]. The natural areas where zebrafish live (the Indian subcontinent and adjacent areas) have a monsoon climate with wide variations in temperature, from 16 to 38°C, and daily oscillations of up to 5.6°C [11]. In our trial, we used 24°C and 28°C as constant experimental temperatures, the latter one being the standard for rearing zebrafish, but we did not observe differences in sex ratio between them, as at both constant temperatures we obtained a major proportion of males. Slightly male-biased sex ratios in cultured zebrafish appear to be common since they have been observed in many facilities around the world. In contrast, both daily thermocycles resulted in a significantly higher proportion of females, which indicates this species is more sensitive to temperature changes than constant temperatures during early development. This finding is supported by the striking differences found in the expression of *cyp19a* and *amh* (Figure 8). Thermocycles acted as a female-promoter by increasing *cyp19a* expression (leading presumably to more estrogen formation [63]) and suppressing *amh* expression in the gonads, resulting in feminization. Proper female sex differentiation in the zebrafish not only depends on germ cells [64] but also on the presence of a certain number of premeiotic germ cells before meiosis can start. In females this process takes place earlier than in males, thus if light regulates the zebrafish cell cycle [41], it could be argued that TC increases or facilitates germ cell proliferation leading to the observed higher proportion of females. However, this would not explain the higher number of females also observed under CT. In any case, it seems clear that cycling temperatures rather than constant ones promote female development. The effects of thermocycles on zebrafish sex ratios presented in this study is consistent with recent finding in Senegalese sole larvae exposed to daily thermocycles (19:22°C, TC), which also produced a larger proportion of females than the constant temperature (20.5°C [65]). In that report, however, reversed thermocycles (22:19°C, CT) led to a larger proportion of males, indicating that temperature effects may be species-dependent. Understanding of the generalities and species-specific differences in relation to sex determination/differentiation in fish is still incomplete. However, current research findings point to the need to integrate not just the influence of environmental factors but also of *cycling* environmental factors and their relationship with the development of the biological clock. Actually, in mammals, clock genes have recently been found to regulate steroid production and aromatase expression in ovarian granulosa cells [66].

In summary, our findings revealed the high sensitivity of zebrafish to temperature and light cycles during early development, and for the first time in this species it is shown that daily thermocycles have multiple and irreversible effects in developing embryos and larvae that ultimately shape the adult phenotype including the sexual phenotype.
